# Preliminary probiotic and technological characterization of *Pediococcus pentosaceus* strain KID7 and *in vivo* assessment of its cholesterol-lowering activity

**DOI:** 10.3389/fmicb.2015.00768

**Published:** 2015-08-04

**Authors:** Karthiyaini Damodharan, Young Sil Lee, Sasikumar A. Palaniyandi, Seung Hwan Yang, Joo-Won Suh

**Affiliations:** ^1^Division of Biosciences and Bioinformatics, Myongji UniversityYongin, South Korea; ^2^Center for Nutraceutical and Pharmaceutical Materials, Myongji UniversityYongin, South Korea; ^3^Graduate School of Interdisciplinary Program of Biomodulation, College of Natural Science, Myongji UniversityYongin, South Korea

**Keywords:** *Pediococcus*, probiotic, oro-gastrointestinal transit, cholesterol-lowering, bile salt hydrolase, LDL-receptor, cholesterol 7**α**-hydroxylase, storage viability

## Abstract

The study was aimed to characterize the probiotic properties of a *Pediococcus pentosaceus* strain, KID7, by *in vitro* and *in vivo* studies. The strain possessed tolerance to oro-gastrointestinal transit, adherence to the Caco-2 cell line, and antimicrobial activity. KID7 exhibited bile salt hydrolase activity and cholesterol-lowering activity, *in vitro*. *In vivo* cholesterol-lowering activity of KID7 was studied using atherogenic diet-fed hypercholesterolemic mice. The experimental animals (C57BL/6J mice) were divided into 4 groups viz., normal diet-fed group (NCD), atherogenic diet-fed group (HCD), atherogenic diet- and KID7-fed group (HCD-KID7), and atherogenic diet- and *Lactobacillus acidophilus* ATCC 43121-fed group (HCD-*L.ac*) as positive control. Serum total cholesterol (T-CHO) level was significantly decreased by 19.8% in the HCD-KID7 group (*P* < 0.05), but not in the HCD-*L.ac* group compared with the HCD group. LDL cholesterol levels in both HCD-KID7 and HCD-*L.ac* groups were decreased by 35.5 and 38.7%, respectively, compared with HCD group (both, *P* < 0.05). Glutamyl pyruvic transaminase (GPT) level was significantly lower in the HCD-KID7 and HCD-*L.ac* groups compared to HCD group and was equivalent to that of the NCD group. Liver T-CHO levels in the HCD-KID7 group were reduced significantly compared with the HCD group (*P* < 0.05) but not in the HCD-*L.ac* group. Analysis of expression of genes associated with lipid metabolism in liver showed that low-density lipoprotein receptor (*LDLR*), cholesterol-7α-hydroxylase (*CYP7A1*) and apolipoprotein E (*APOE*) mRNA expression was significantly increase in the HCD-KID7 group compared to the HCD group. Furthermore, KID7 exhibited desired viability under freeze-drying and subsequent storage conditions with a combination of skim milk and galactomannan. *P. pentosaceus* KID7 could be a potential probiotic strain, which can be used to develop cholesterol-lowering functional food after appropriate human clinical trials.

## Introduction

High blood cholesterol (hypercholesterolemia) is a risk factor for cardiovascular diseases, which remains one of the largest causes of death worldwide (Ishimwe et al., 2015). It has been shown that a 1% reduction in serum cholesterol is associated with an estimated reduction of 2–3% in the risk of coronary heart disease (CHD) (Ishimwe et al., 2015). Statin drugs are predominantly prescribed for the reduction of serum cholesterol level and in turn the risk of CHD. There is an increasing interest in non-drug therapies for lowering serum cholesterol and the risk of CHD due to several adverse side effects of statin drugs reported in the literature and by patients (Sultan and Hynes, 2013). An alternative is probiotics with cholesterol-lowering activity. Probiotics are defined as “live microorganisms that, when administered in adequate amounts, confer a health benefit on the host” (Hill et al., 2014). Recently, several studies have shown that probiotics could be used as alternative supplements to exert cholesterol-lowering effects in humans (Trautvetter et al., 2012; Fuentes et al., 2013; Guardamagna et al., 2014). Probiotics have been suggested to reduce cholesterol via various mechanisms (Ishimwe et al., 2015). Available literature suggests that probiotics with bile salt hydrolase (BSH) activity show cholesterol-lowering activity *in vivo* (Kumar et al., 2011; Jones et al., 2012, 2014; Pavlovic et al., 2012; Degirolamo et al., 2014). Other mechanisms such as cholesterol adsorption to cell surface, cholesterol assimilation into bacterial cell membrane (Liong and Shah, 2005a) and co-precipitation with deconjugated bile acids (Liong and Shah, 2005b) are also proposed/demonstrated *in vitro* without evidence of their occurrence *in vivo*.

Several species and strains belonging to the orders *Lactobacillales* and *Bifidobacteriales* are widely recognized, approved and used as probiotics with some yeast and *Bacillus* sp. as well. The beneficial effects of probiotics include but are not limited to gastro-intestinal microbial balance, suppression of pathogens, immunomodulatory activity, hypocholesterolemic activity, and alleviation of certain conditions such as diarrhea, allergy, lactose intolerance, irritable bowel syndrome, inflammatory bowel disease (IBD), and colon cancer (reviewed in Nagpal et al., 2012). However, a single probiotic microbe cannot provide all of these beneficial effects and efficacy and the activity of probiotic strains vary considerably. For example, certain probiotic strains show efficacy against antibiotic-associated diarrhea but there is less evidence for their efficacy against IBD.

Since, the probiotic property of microbes differs from one strain to another, new strains must be assessed for their putative probiotic properties according to FAO/WHO guidelines (FAO-WHO, 2002). The FAO/WHO guideline recommends certain testing methods to establish the health benefits of a microbe to be called probiotic. The testing methods include *in vitro* and *in vivo* study of oro-gastrointestinal transit tolerance, production of antimicrobial substances, beneficial probiotic characters such as cholesterol-lowering activity, anti-hypertensive activity, anti-diabetic activity etc., and adherence to human intestinal cells, before testing the microbe in human clinical trials (FAO-WHO, 2002). The guidelines also insist on the characterization of a putative probiotic microbe for its safety that the strain should not possess any transferrable antibiotic resistance (FAO-WHO, 2002). Additionally, any probiotic microbe should maintain its viability and probiotic activity during industrial manufacturing practices such as drying, and storage in various products.

The objective of this study was to establish the various probiotic properties and cholesterol-lowering activity of a *Pediococcus pentosaceus* strain KID7, through *in vitro* and *in vivo* studies and technological characterization of strain KID7 for its ability to maintain desired viability during manufacturing and storage condition.

## Materials and methods

### Microorganisms and culture conditions

Strain KID7 was isolated from fermented finger millet (*Eleusine coracana*) gruel obtained from a household in Yongin, Korea. The gruel was serial-10-fold diluted and 10^−4^ to 10^−6^ dilutions were plated on de Man Rogosa and Sharpe (MRS) agar medium and incubated at 37°C for 24 h to pick as single colony of strain KID7. The probiotic strains *Lactobacillus rhamnosus* GG and *Lactobacillus acidophilus* ATCC 43121 were obtained from American Type Culture Collection (ATCC, Manassas, VA, USA) and the type strain *Pediococcus pentosaceus* KACC 12311 was obtained from the Korean Agricultural Culture Collection (KACC), Republic of Korea. The strains were cultured in MRS agar medium and incubated at 37°C for 24 h before being used for experiments. The strains were stored as glycerol stocks (20% glycerol in MRS broth) at −80°C. The probiotic strains and type strain *P. pentosaceus* KACC 12311 were used as reference strains for comparison of probiotic and biochemical characteristics, respectively.

Pathogenic microbes used in this study were obtained from the Korean Collection for Type Cultures (KCTC), KACC and Korean Culture Center of Microorganisms (KCCM), Republic of Korea. The pathogenic strains were routinely cultured in Luria-Bertani (LB) agar medium and stored as glycerol stocks (20% glycerol in LB broth, v/v) at −80°C.

### Strain identification and biochemical test

KID7 was identified by gram staining, microscopic examination and the API 50 CHL kit (Biomerieux S.A., La Balme les Grottes, France). In addition, the fermentation pattern of KID7 was identified using homo- and hetero-fermentation (HHD) medium (Mcdonald et al., 1987). Enzyme activities such as β-galactosidase, β-glucosidase and protease activity were studied following previous reports (Vidhyasagar and Jeevaratnam, 2013; Lee et al., 2014). KID7 was studied for its growth in the presence of NaCl (1–10%, w/v) in MRS broth. Genomic DNA of strain KID7 was isolated using a genomic DNA isolation kit (GeneALL, Seoul, South Korea), following the manufacturer's protocol. PCR amplification of the 16S rRNA gene from KID7 was performed with the primers 27F and 1492R (Borges et al., 2013) and sequencing of the PCR product was done with 27F and 785F (5′-GGATTAGATACCCTGGTA-3′) primers to get a partial sequence of the 16S rRNA gene. Sequencing service was provided by Solgent Co. Ltd. (Seoul, South Korea). The sequence was searched for similarities in the EzTaxon database using the BLAST program. A phylogenetic tree was constructed using the closely related sequences by multiple alignment using Clustal X followed by neighbor-joining phylogenetic tree construction using MEGA 6 software (Tamura et al., 2013).

#### *In vitro* study of probiotic properties

##### Safety assessment

KID7 was subjected to safety assessment such as biogenic amine production, hemolytic activity, and degradation of type III mucin from porcine stomach (Sigma, St. Louis, MO, USA). The biogenic amine production was tested according to the procedure described by Bover-Cid and Holzapfel (1999) using decarboxylase agar medium with or without amino acids such as L-phenylalanine, L-lysine, L-tryptophan, L-tyrosine, L-arginine, L-ornithine, or L-histidine. Hemolytic activity and mucin degradation were tested by petri dish-based methods described by Borges and Teixeira (2014) and Zhou et al. (2001), respectively. The minimum inhibitory concentration (MIC) of various antibiotics for strain KID7 was tested by 2-fold broth microdilution method (Wiegand et al., 2008). Susceptibility of the strain to a particular antibiotic was determined according to the cut-off MIC values given by European Food Safety Authority (EFSA, 2012).

##### Oro-gastrointestinal transit assay

Oro-gastrointestinal transit (OGT) tolerance assay was performed according to the method described by Bove et al. (2012) with modification. The assay mimic the physiological conditions of oral, gastric and intestinal stress. The strain KID7 was subjected to oral stress for 10 min in electrolyte solution (sodium chloride, 6.2 g; potassium chloride, 2.2 g; calcium chloride, 0.22 g; sodium bicarbonate, 1.2 g) (Marteau et al., 1997) with 150 mg/L lysozyme (Sigma). This was followed by gastric stress for a 30-min incubation in electrolyte solution of pH 3+0.3% pepsin (Sigma) and another 30-min incubation in electrolyte solution of pH 2+0.3% pepsin. Finally, the cells were subjected to a 120-min incubation in intestinal electrolyte solution (sodium chloride, 5 g; potassium chloride, 0.6 g; calcium chloride, 0.25 g; pH 7) (Marteau et al., 1997) with 0.3% bile oxgall (BD) and 0.1% pancreatin (Sigma-Aldrich). The strains in PBS without stress were used as control. The cells were separated from the stress solution after each stress by centrifugation at 5000 rpm for 5 min and either subjected to next stress or used for viability analysis. The viability was calculated from colony-forming units (cfu) of appropriate dilutions from the control and stress-treated bacterial cells plated on MRS agar medium incubated for 48 h at 37°C. The bacterial viability at the end of each stress was also monitored using the LIVE/DEAD BacLight bacterial viability kit (Invitrogen, Oregon, USA) following the manufacturer's protocol. The live and dead bacteria were visualized as green and red fluorescent cells, respectively under a fluorescence microscope (Olympus BX50, Tokyo, Japan) and photomicrographs were captured using a digital camera (Olympus DP70, Tokyo, Japan).

##### Caco-2 cell culture and adhesion assay

Caco-2 cell line was obtained from KCTC (Korea) and routinely cultured in minimum essential medium (MEM) high-glucose medium (Gibco) supplemented with 20% (v/v) inactivated fetal bovine serum and 100 U of antibiotics penicillin-streptomycin. The culture was incubated at 37°C with 5% CO_2_. For theadhesion assay, the Caco-2 cell line was used between the 39th and 41st passage, which phenotypically resembled the enterocytes and expressed tight junction. The Caco-2 cells were seeded at the concentration of 2 × 10^5^ cfu/ml in 12-well cell culture plates and incubated for 21 days to get polarized monolayer and 100% confluence. The medium was changed every day.

The adhesion of lactic acid bacteria (LAB) strains to Caco-2 cells was determined by the method described by Lee et al. (2014) with minor modification. Briefly, the Caco-2 cells were replaced with non-antibiotic-supplemented MEM medium 2 h prior to the adhesion assay. 0.5 ml of 18-h bacterial suspension in PBS was mixed with an equal volume of non-antibiotic-supplemented cell culture medium. The final concentration of bacteria was 1 × 10^8^ cfu/ml, added to each well of the tissue culture plate containing the Caco-2 monolayer, and incubated at 37°C in 5% CO_2_ for 2 h. After incubation, the number of adhered bacterial cells was determined according to Lee et al. (2014).

##### Antimicrobial activity

The antimicrobial activity of strain KID7 was determined against human, animal and food-borne pathogens listed in **Table 2**. Strain KID7 was cultured in MRS broth for 48 h at 37°C and the culture filtrate was concentrated 10-fold by lyophilization. One hundred microliter of concentrated crude culture filtrate was loaded on to an 8-mm paper disc (ADVANTEC, Japan) and dried. The disc was placed on the pathogen- spread LB agar plate and incubated for 18 h and the zone of inhibition was measured. The entire assay was performed in triplicates to check the reproducibility. Hydrogen peroxide production by the LAB strains was determined according to the method of Saito et al. (2007).

##### Bile salt hydrolase activity

Bile salt hydrolase activity of KID7 was detected by thin-layer chromatography (TLC). The reaction mixtures were prepared by a method described by Guo et al. (2011) with slight modification. MRS reaction mixture was prepared with 5 mM taurodeoxycholic acid (TDCA), or 5 mM glycocholic acid (GCA), or 5 mM taurocholic acid (TCA) in sodium phosphate buffer (pH 7.4). One milliliter of 18-h culture was mixed with an equal volume of phosphate-buffered MRS + conjugated bile acid and incubated for 36 h at 37°C. After incubation, the mixture was lyophilized, dissolved in 1 ml of methanol and centrifuged to remove precipitates. Three microliter of sample was spotted on TLC (silica gel 60, 20 × 20 cm) and developed using a mobile phase of hexane:methylethlyketone:glacial acetic acid (56:36:8, v/v/v) (Chavez and Krone, 1976). After development, the plates were dried with hot air, sprayed with 10% sulfuric acid in distilled water, and heated at 110°C for 10 min in a hot air oven to detect the free cholic acid, deoxycholic acid, and conjugated bile acids. The spots were visualized under UV illumination.

##### Determination of cholesterol-lowering activity

The LAB strains were studied for cholesterol-lowering activity in MRS liquid broth supplied with cholesterol. MRS broth was initially prepared with 0.05% cysteine-HCl to create microaerophilic condition and autoclaved, followed by addition of cholesterol (CHO) dissolved in ethanol:Tween 80 (3:1, v/v) to a final concentration of 500 μg/ml, w/v, in the MRS medium. The ethanol concentration should not exceed 5%. The MRS-CHO broth was inoculated with log phase bacterial culture (2% v/v of A_600_ = 0.5 OD culture) and incubated at 37°C37°C for 24 h. After the incubation, bacterial cells were pelleted out by centrifugation at 1500 × g at 4°C4°C for 10 min. The cholesterol concentration in spent medium was estimated by enzymatic method using the BCS total cholesterol kit (Bioclinical System, Seoul, South Korea) following the manufacturer's protocol and compared with uninoculated MRS-CHO broth as control and the percentage cholesterol removal was calculated. The cholesterol-lowering activity of LAB was also studied in the presence of 0.3 and 0.5% bile. Additionally, growth of the LAB strains was also measured in MRS-CHO, and MRS-CHO-bile (0.3 and 0.5%) broth.

#### *In vivo* study of cholesterol-lowering activity of KID7

##### Experimental animals

Male C57BL/6J mice (7–8 weeks-old) were purchased from Orient Bio (Sung-Nam, South Korea). The mice were housed under temperature (23 ± 3°C) and humidity-controlled conditions (40 ± 6%) with a 12-h light/dark cycle and were given free access to water and food throughout the experiment. After acclimatization, the mice were fed an atherogenic diet (HCD, 40 kcal % fat, 1.25% cholesterol and 0.5% cholic acid, Cat #101556; Research Diets, NJ, USA) for 4 weeks to induce hypercholesterolemia before the oral administration of LAB. One group was continued with a normal diet (NCD). After 4 weeks, HCD-fed mice were assigned to one of the following three groups for 4–5 weeks: HCD-control (Con); HCD administered with 3 × 10^8^ cfu/ml of KID7 (HCD-KID7); or HCD administered with 3 × 10^8^ cfu/ml of *L. acidophilus* ATCC 43121 (HCD-*L.ac*). KID7 and *L. acidophilus* ATCC 43121 were mixed with distilled water and administered by oral gavage once daily for 32 days and the control NCD and HCD groups were administered distilled water alone. The body weight was measured once per week for 6 weeks. Food intake (g/mouse/day) was determined by subtracting the remaining food weight from the initial food weight of the previous feeding day, and dividing by the number of mice housed in the cage. The experimental design was approved by the Ethical Committee of the Myongji Bioeffeciency Research Center in Myongji University (Yongin-Si, Gyeonggi-do, Republic of Korea), and the mice were maintained in accordance with standard guidelines.

##### Collection of tissues

At the end of the 32-days treatment period, the mice were sacrificed by cervical dislocation, and the liver and white adipose tissue (WAT) were excised immediately, rinsed, weighed, frozen in liquid nitrogen, and stored at −80°C until analysis.

##### Serum biochemical analysis

For biochemical analyses of serum, blood samples were collected from tail vein at 16-h-fasted state. Serum was obtained from the blood by centrifugation (3000 rpm, 10 min, 4°C) and was stored at −80°C until analysis. Serum total cholesterol (T-CHO), triglyceride (TG), HDL-cholesterol, glucose, glutamyl oxaloacetic transaminase (GOT) and glutamyl pyruvic transaminase (GPT) levels were determined by using commercial assay kits (Asan Pharm, Seoul, Korea). Low-density lipoprotein (LDL) cholesterol was calculated using the Friedewald formula (Friedewald et al., 1972).

##### Liver and fecal lipid contents

Lipids from the isolated liver and feces was extracted using the Folch method (Folch et al., 1957). The extracted lipid fraction was used to measure the T-CHO and TG levels using commercial kit mentioned above. Fecal cholic acid content was measured following the method of Kumar et al. (2011).

##### Total RNA isolation and gene expression analysis

Total RNA was extracted from liver using the RNeasy mini kit (Qiagen Korea, Seoul, South Korea) according to the manufacturer's protocol. Total RNA (1 μg) was reverse-transcribed to cDNA with the PrimeScript RT reagent kit (Takara, Shiga, Japan). The mRNA expression levels of cholesterol metabolisms-related genes were evaluated by real-time PCR using iQTM SYBR Green Supermix and the real-time PCR iQTM5 system (Bio-Rad Laboratories, Hercules, CA, USA). Relative gene expression levels were calculated by the difference in Ct value after normalization with β-actin (*ACTB*) and were expressed as the fold-change relative to the HCD control group. The primers used in the experiments are listed in Table 1.

**Table 1:** **The primer sequences used for quantitative real-time PCR**.

**Gene name**	**Forward (5^′^ → 3^′^)**	**Reverse (5^′^ → 3^′^)**	**Reference**
*SREBF2*	GGCCTCTCCTTTAACCCCTT	CACCATTTACCAGCCACAGG	Ahn et al., 2013
*LDLR*	GCGTATCTGTGGCTGACACC	TGTCCACACCATTCAAACCC	
*HMGCR*	GGGACCAACCTTCTACCTC	GCCATCACAGTGCCACATA	Wang et al., 2014
*CYP7A1*	TCTCAACGATACACTCTCCACC	CTTCAGAGGCTGCTTTCATTGC	
*APOE*	GAACCGCTTCTGGGATTACCT	TCAGTGCCGTCAGTTCTTGTG	Ding et al., 2012
*ACTB*	TGTCCACCTTCCAGCAGATGT	AGCTCAGTAACAGTCCGCCTAGA	

*ACTB, β-actin; SREBF2, sterol regulatory element binding protein 2; HMGCR, 3-hydroxy-3-methylglutaryl-CoA reductase; LDLR, Low-density lipoprotein receptor; CYP7A1, cytochrome P450 family 7 subfamily A polypeptide 1 (cholesterol 7 α-hydroxylase); APOE, apolipoprotein E*.

#### Technological characterization

##### Viability of strain KID7 under freeze-drying and storage conditions

KID7 was grown in MRS broth at 37°C for 30 h (late log-phase culture) and the cells were harvested by centrifugation at 3000 × g for 15 min at 4°C. The cell pellet was washed and resuspended in PBS (control) and additives to give a final cell concentration of 1.8 × 10^9^ cfu/ml. The additives used were 1% (w/v) skim milk, 1% (w/v) galactomannan (Sigma-Aldrich), 1% (w/v) wheat bran hydrolysate, 0.5% (w/v) skim milk + 0.5% (w/v) galactomannan, and 0.5% (w/v) galactomannan + 0.5% (w/v) wheat bran hydrolysate, prepared separately in sterile distilled water. Wheat bran hydrolysate was prepared following the method of Wang et al. (2009). The cell suspension in PBS and additives were frozen at −80°C. The frozen suspensions were subjected to freeze-drying using a lab-scale freeze dryer (IlShin lab Co., ltd, Korea), pre-cooled to −80°C. The samples were dried under reduced pressure (5 mbar) for 24 h. The freeze-dried samples were rehydrated using sterile distilled water and serially diluted and plated on MRS agar to determine the viable count. Alternatively, the freeze-dried samples were stored at three different temperature viz., −20, 4, and 25°C for up to 6 months. The viable cell count present in each sample was determined as described above. The number of colonies on MRS agar was calculated and the total cfu was calculated for each sample stored at various temperatures and time points.

##### Statistical analysis

Data are expressed as means ± SEM. The differences among treatment groups were analyzed by One-Way ANOVA, followed by Duncan test, using Origin 7 Software (MicroCal Software, Northampton, MA, USA). Values of *P* < 0.05 were considered to indicate statistical significance.

## Results

### Identification of strain KID7 based on 16S rDNA sequence and biochemical test

BLAST search of the EzTaxon database showed that the 16S rDNA sequence of KID7 (Genbank accession number KJ810576) (1508 bp) showed 99.3% (difference of 10/1499 bp) similarity with *P. pentosaceus* type strain DSM 20336. All other *Pediococcus* species showed less than 98.5%, which is the cut-off value for species-level comparison studies (Stackebrandt, 2011). Phylogenetic analysis of the 16S rRNA gene sequence of KID7 with that of various *Pediococcus* species confirmed its close relatedness to *P. pentosaceus* DSM 20336 (Figure S1). Hence, *P. pentosaceus* KACC 12311 (=DSM 20336) was used for comparative biochemical test with KID7. The biochemical tests of strain KID7 and the closely related strain *P. pentosaceus* KACC 12311 are shown in Table S1. The strain KID7 possess various enzymatic activities such as protease, β-galactosidase and β-glucosidase similar to that of *P. pentosaceus* KACC 12311. It showed difference in the carbohydrate utilization pattern, which is considered as strain-level difference between KID7 and *P. pentosaceus* KACC 12311. Strain KID7 grew well in the presence of NaCl, however, a reduction in cell growth was observed with increasing concentration of NaCl (Figure S2).

#### *In vitro* probiotic properties of KID7

##### *In vitro* safety assessment of KID7

Strain KID7 was negative for hemolytic activity on blood agar (Figure S3B), mucin degradation on mucin-supplied medium (Figure S3C), and bioamine production on various amino acid-supplied decarboxylase agar media (Figure S3A). The minimum inhibitory concentration (MIC) of strain KID7 for selected antibiotics was shown in Table 2. The strain KID7 was sensitive to all antibiotics except kanamycin B sulfate and streptomycin sulfate.

**Table 2:** **Antibiotic susceptibility of ***P. pentosaceus*** KID7**.

**Antibiotics**	**Minimum inhibitory concentration (MIC) (mg/L)**	**EFSA cut-off (MIC) (mg/L)**
Chloramphenicol	1 (S)	4
Tetracyclin hydrochloride	4 (S)	8
Erythromycin	0.25 (S)	1
Streptomycin sulfate	256 (R)	64
Gentamycin C	8 (S)	16
Clindamycin	0.5 (S)	1
Ampicillin	1 (S)	4
Kanamycin B sulfate	1024 (R)	64

*Bacterial susceptibility to antibiotic was determined according to the cut-off values given by EFSA (2012). S, Susceptible; R, Resistant*.

##### Survival of KID7 under simulated oro-gastrointestinal transit condition

**Figure 1:**
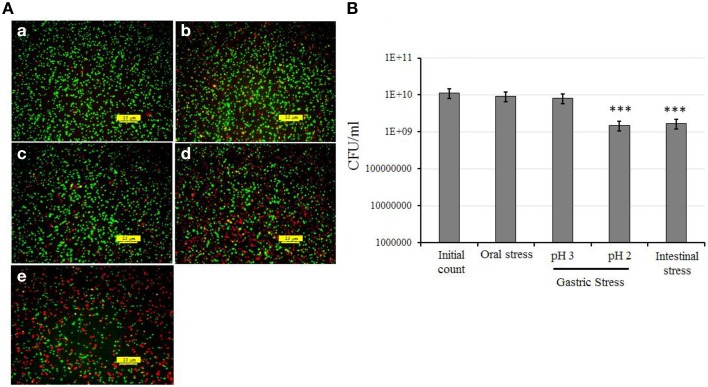
*****In vitro*** assessment of oro-gastrointestinal transit tolerance of strain KID7 under simulated conditions. (A)** Fluorescence photomicrograph of KID7 cells after various stages of simulated oro-gastrointestinal transit: (a) initial, (b) oral stress, (c) gastric stress pH 3, (d) gastric stress pH 2, and (e) intestinal stress. The live, and dead/damaged cells were identified by using the LIVE/DEAD BacLight bacterial viability kit after oral, gastric and intestinal stress treatment. Green color indicates viable bacteria; red color indicates damaged or dead bacteria. **(B)** Viable count of KID7 after each stages of simulated oro-gastrointestinal transit. The viable count of KID7 was determined by plating the cells from each stage on MRS agar and incubation at 37°C for 48 h. Error bar represents standard error of mean of three independent experiments. ^***^ Significantly different from initial count at *P* < 0.005.

The strain KID7 showed good tolerance to oral, gastric and intestinal stress based on the results of fluorescence microscopic analysis for live and dead/damaged cells (Figure 1A) and plate count obtained after each stress treatment (Figure 1B). We observed a high amount of red fluorescent cells (indicating dead/damaged cells) in the gastric stress (pH 2) and intestinal stress in the fluorescence microscopic analysis (Figure 1A). However, we observed that these were only damaged cells, as they recovered during culture on MRS agar, which was evidenced by the number of colony-forming units in the gastric stress and intestinal stress (Figure 1B). Overall, a reduction of 1 log unit cfu (*P* < 0.05) was observed at the end of the OGT assay compared to the initial count (Figure 1B).

##### Adherence of KID7 to Caco-2 cell monolayer

**Figure 2:**
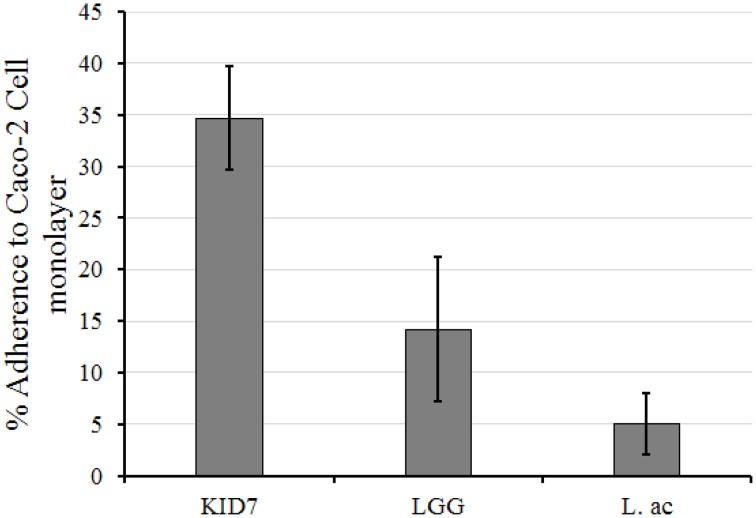
**Adherence of ***P. pentosaceus*** strain KID7, ***L. rhamnosus*** GG, and ***L. acidophilus*** ATCC 43121 to Caco-2 cell monolayer**. The error bars indicate standard deviation of three independent experiments.

The adherence of KID7, *L. rhamnosus* GG, and *L. acidophilus* ATCC 43121 to Caco-2 cell monolayer was calculated as the percentage of cells that remained attached to the Caco-2 monolayer after two washes compared to the initial amount of LAB added (Figure 2). Plate count of the attached bacterial cells revealed high adherence of KID7 cells (34.7%) (Figure 2) compared to *L. rhamnosus* GG (14.2%) and *L. acidophilus* ATCC 43121 (5%) (Figure 2).

##### Antimicrobial activity of strain KID7

The concentrated culture filtrate from KID7 showed a broad-spectrum antimicrobial activity against human, animal and food-borne pathogens used in this study (Table 3). The antimicrobial activity was observed against both gram-positive and gram-negative pathogenic bacteria, whereas no antimicrobial activity was observed against LAB strains tested. We observed that KID7 (Figure S4A) and the reference strain *P. pentosaceus* KACC 12311 (Figure S4B) did not produce H_2_O_2_, whereas, *L. rhamnosus* GG produces H_2_O_2_, which is observed as a zone of blue color around the colony on Prussian blue agar (Figure S4C).

**Table 3:** **Antimicrobial activity of culture filtrate of strain KID7**.

**Test strains**	Zone of inhibition (mm)^*^
**GRAM POSITIVE PATHOGENS**	
*Staphylococcus aureus* KCCM 11335	15
*Staphylococcus epidermidis* KCTC 1917	12
*Listeria monocytogenes* KACC 10764	20
*Staphylococcus aureus* KCCM 40510 (Methicillin- resistant)	11
*Bacillus cereus* KACC 11240	10
**GRAM NEGATIVE PATHOGENS**	
*Salmonella typhi* KCTC 2514	12
*Salmonella choleraesuis* KCTC 2932	15
*Pseudomonas aeruginosa* KCCM 11802	18
*Yersinia enterocolitica* ssp. *enterocolitica* KACC 15320	20
*Salmonella gallinarum* KCTC 2931	26
*Shigella boydii* KACC 10792	14
*Escherichia coli* O138 KCTC 2615	11
*E. coli* O1 KCTC 2441	12
**LACTIC ACID BACTERIA (GRAM POSITIVE)**	
*Enterococcus durans* KACC 10787	–
*Lactobacillus fermentum* KACC 11441	–
*Lactobacillus plantarum* ssp. *plantarum* KACC 11451	–
*Lactobacillus brevis* KACC 11433	–
*Lactobacillus paracasei* KACC 12361	–

* *Concentrated crude culture filtrate of strain KID7 was used for the antimicrobial assay. –, Indicates no zone of inhibition*.

##### Bile salt hydrolase and cholesterol-lowering activity of KID7

**Figure 3:**
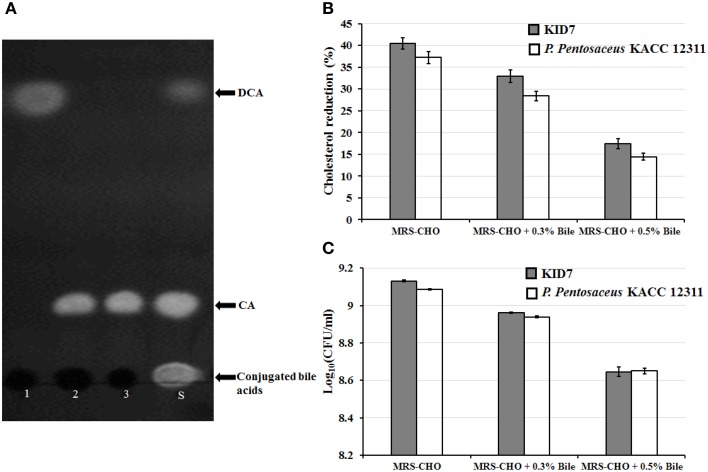
*****In vitro*** cholesterol-lowering activity of ***P. pentosaceus*** KID7. (A)** Thin-layer chromatography analysis of free cholic acid present in the culture filtrate of KID7 grown in MRS containing various conjugated bile salts. 1, MRS+5 mM TDCA; 2, MRS+5 mM TCA; 3, MRS+5 mM GCA; S, Standard mixture of TDCA, TCA, GCA, cholic acid (CA), and deoxycholic acid (DCA). **(B)** Cholesterol reduction activity of strain KID7 and *P. pentosaceus* KACC 12311 with or without bile-oxgall. **(C)** Growth of KID7 and *P. pentosaceus* KACC 12311 in MRS-CHO medium with or without bile-oxgall represented in log cfu/ml. The data are representation of mean values of three independent assays and error bars indicates ± standard deviation.

The strain KID7 possessed bile salt hydrolase activity that deconjugated TDCA, GCA and TCA (Figure 3A). We observed a 40% decrease in *in vitro* cholesterol concentration in MRS-CHO broth after the growth of KID7 for 24 h. However, this reduction level was decreased in the presence of bile oxgal—0.3% (33% cholesterol reduction) and—0.5% (17% cholesterol reduction) (Figure 3B). The percentage of cholesterol reduction by KID7 was comparatively higher than that of *P. pentosaceus* KACC 12311 (Figure 3B). The reduction in cholesterol-lowering activity of KID7 and *P. pentosaceus* KACC 12311 in the presence of 0.3 and 0.5% bile oxgal may be due to reduced cell growth in the presence of bile compared to bile- non-supplied MRS-CHO broth (Figure 3C). These observations suggest that cell concentration is the key factor that affects cholesterol reduction in liquid broth.

#### *In vivo* cholesterol-lowering activity of KID7

##### Effects of KID7 and *L. acidophilus* on body weight gain, food intake, and organ weight

The body weight gain, food intake, and organ weight are shown in Table 4. Body weight gain was not significantly different among all groups. Food intake of the NCD group was higher than that of the HCD control group (*P* < 0.005), but there was no significant difference in food intake among the HCD control and HCD-LAB groups. WAT weight did not differ among all groups. Liver weights in the NCD group were reduced vs. that of the HCD control group, but did not differ in the HCD control and HCD-LAB groups.

**Table 4:** **Effects of KID7 and ***L. acidophilus*** on body weight, food intake, and tissues weight in mice**.

**Parameters**	**Dietary groups**			
	**NCD**	**HCD**	**HCD /+ KID7**	**HCD /+ L. acidophilus**
**BODY WEIGHT (g)**				
Initial	21.77 ± 0.48	22.24 ± 0.49	20.89 ± 0.52	21.03 ± 0.37
Final	23.24 ± 0.49	22.01 ± 0.36	21.97 ± 0.50	22.64 ± 0.45
Gain	1.88 ± 0.35	1.56 ± 0.39	1.68 ± 0.40	1.8 ± 0.70
Food intake rate (g/mouse/day)	3.24 ± 0.18	2.50 ± 0.23	2.5 ± 0.21	2.42 ± 0.17
**TISSUES WEIGHT (g)**				
Liver	0.9 ± 0.03^***^	1.29 ± 0.05	1.27 ± 0.04	1.21 ± 0.52
Epididymal fat	0.16 ± 0.03	0.16 ± 0.019	0.17 ± 0.015	0.20 ± 0.033

NCD, Normal diet; HCD, high-cholesterol diet; KID7, P. pentosaceus KID7; L.ac, L. acidophilus. Data are expressed as means ± SEM (n = 8/group).

*** *Significantly different at P < 0.005 vs. the HCD group*.

##### Effects of KID7 and *L. acidophilus* on the serum biochemical levels

**Figure 4:**
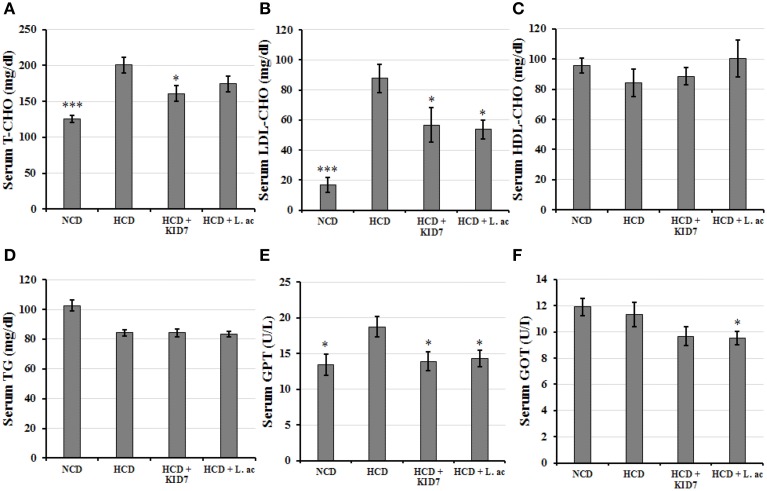
**Effects of KID7 and ***L. acidophilus*** on the serum biochemical parameters**. Mice were fed either normal diet, atherogenic diet, or atherogenic diet supplemented with KID7 or *L. acidophilus* for 32 days. **(A)** T- CHO; **(B)** LDL-CHO; **(C)** HDL-CHO; **(D)** TG; **(E)** GPT; **(F)** GOT. NCD: normal diet; HCD: high-cholesterol diet; HCD-KID7: HCD + *P. pentosaceus* KID7; HCD-*L.ac*: HCD + *L. acidophilus*. Data are expressed as means ± SEM (*n* = 8/group). ^*^, ^***^ indicate significantly lower at *P* < 0.05 and *P* < 0.005, respectively, vs. the HCD group.

As shown in Figure 4, T-CHO and LDL-CHO levels in the NCD group were significantly lower compared to the HCD control group (all, *P* < 0.005). T-CHO levels were significantly decreased by 19.8% in the HCD-KID7 group (*P* < 0.05), but did not in the HCD-*L.ac* group (Figure 4A). LDL-CHO levels in both the HCD-KID7 and HCD-*L.ac* group were decreased by an average of 35.5 and 38.7%, respectively, compared with the HCD control group (both, *P* < 0.05) (Figure 4B). HDL-CHO and TG level gain was not significantly different among all groups (Figures 4C,D). GPT levels in the NCD, HCD-KID7, and HCD-*L.ac* groups were reduced by 30, 30, and 28%, respectively, vs. the HCD control group (all, *P* < 0.05) (Figure 4E). GOT levels in the HCD-KID7 and HCD-*L.ac* groups showed the tendency to reduce, but not statistically significant (Figure 4F).

##### Effects of KID7 and *L. acidophilus* on lipid content in liver and feces

**Figure 5:**
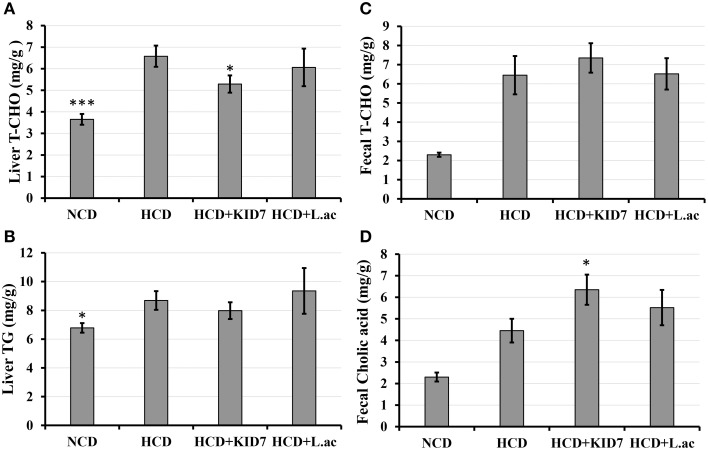
**Effects of ***P. pentosaceus*** KID7 and ***L. acidophilus*** on hepatic and fecal lipid levels**. Mice were fed either normal diet, atherogenic diet or atherogenic diet supplemented with KID7 or *L. acidophilus* for 32 days. **(A)** Liver T-CHO; **(B)** Liver TG; **(C)** Fecal T-CHO, and **(D)** Fecal cholic acid contents. NCD: normal diet; HCD: high-cholesterol diet; HCD-KID7: HCD + *P. pentosaceus* KID7; HCD-*L.ac*: HCD + *L. acidophilus*. Data are expressed as means ± SEM (*n* = 8/group). ^*^, ^***^ indicate significantly different (lower for **A** and **B**, higher for **C** and **D**) at *P* < 0.05 and *P* < 0.005, respectively, vs. the HCD group.

Liver T-CHO levels and TG in the NCD group were lower than that of the HCD control group (*P* < 0.001 and *P* < 0.05, respectively) Figure 5. Liver T-CHO levels in the HCD-KID7 group were reduced significantly compared with the HCD control group (*P* < 0.05) (Figure 5A). Liver TG levels tended to decrease in the HCD-KID7 group compared with the HCD control group, but not statistically significant (Figure 5B). Fecal T-CHO levels tended to increase in the HCD-KID7 group compared with the HCD control group, but not statistically significant (*P* = 0.056) (Figure 5C). A significant increase (*P* < 0.05) in fecal cholic acid content was observed in the HCD-KID7 group compared to HCD group. In the HCD-*L.ac* group no significant change in liver total cholesterol, liver triglyceride content, fecal cholesterol and fecal cholic acid contents was noted compared to the HCD control group (Figures 5A–D).

##### Effects of KID7 and *L. acidophilus* on expression levels of genes associated with cholesterol metabolism in liver

**Figure 6:**
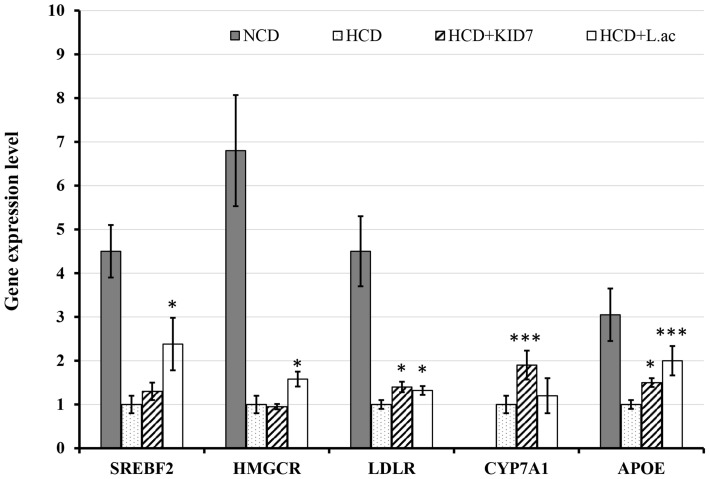
**Effects of KID7 and ***L. acidophilus*** on expression levels of lipid metabolism-related genes in mice liver**. The mRNA expression levels were evaluated by quantitative real-time PCR. Data were normalized to β-actin RNA expression levels and then compared to the HCD group, which was assigned a value of 1.0. NCD: normal diet; HCD: high-cholesterol diet; HCD-KID7: HCD + *P. pentosaceus* KID7; HCD-*L.ac*: HCD + *L. acidophilus*. Data are expressed as means ± SEM (*n* = 8/group). ^*^, ^***^ indicate significantly higher at *P* < 0.05 and *P* < 0.005, respectively, vs. the HCD group.

We examined the expression levels of cholesterol metabolism-related genes to understand the mechanism of action of KID7 compared to *L. acidophilus* ATCC 43121 in improving hypercholesterolemia (Figure 6). The NCD feeding was found to significantly increase the *SREBF2, HMGCR, LDLR*, and *APOE* gene expression levels compared to those of the HCD group. *SREBF2* gene expression level did not change in the HCD-KID7 group, but significantly increased in the HCD-*L.ac* group compared to the HCD group (Figure 6). *LDLR* expression level was significantly increased in the HCD-KID7 and in the HCD-*L.ac* group. Meanwhile, no significant difference was observed in *HMGCR* expression in the HCD-KID7 compared to HCD group (Figure 6). A significant increase in *CYP7A1* mRNA expression level was observed in the HCD-KID7 but not in HCD-*L.ac* group compared to HCD. *APOE* mRNA expression level was significantly increased in the HCD-KID7 and HCD-*L.ac* groups vs. that of the HCD group (Figure 6).

#### Technological characteristic of KID7

##### Viability of KID7 under freeze-drying and storage conditions

**Figure 7:**
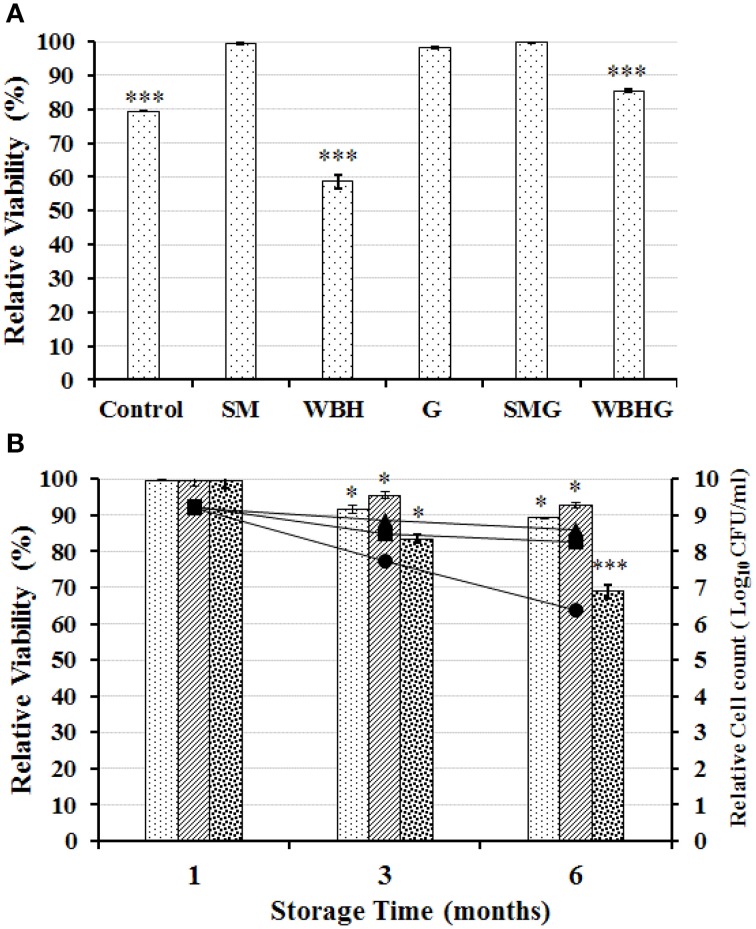
**Viability of strains KID7 under freeze-drying and storage conditions. (A)** Viability of strain KID7 after freeze-drying with various combinations of food grade additives such as SM, skim milk (1%); G, galactomannan (1%); WBH, wheat bran hydrolysate (1%); SMG, skim milk (0.5%) + galactomannan (0.5%); WBHG, wheat bran hydrolysate (0.5%) + galactomannan (0.5%); and Control, KID7 cells in PBS without food grade additives. **(B)** Viability of strain KID7 combined with SMG, skim milk (0.5 %) + galactomannan (0.5%) under different storage conditions such as −20°C (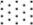, ■), 4°C (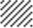, ▴), and 25°C (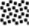, •), up to 6 months. Histogram represents percentage viability of KID7 under different storage condition. Line graph represents relative logarithmic value of colony-forming units of KID7 under different storage condition. Error bars represent standard error of mean of three independent experiments. ^*^P < 0.05 and ^***^P < 0.005 significantly different at the *P*-values compared to initial count.

The viability of strain KID7 after freeze-drying condition in the presence of various food grade additives was given in Figure 7A. Among the tested additives, 0.5% skim milk + 0.5% galactomannan (SMG), protected the bacterial cells during freeze-drying condition and the viability was retained at 99.79%, which was better than in the presence of 1% skim milk alone (viability = 99.6%) (Figure 7A). Further, we tested KID7 cells in SMG combination for storage at different temperature conditions in freeze-dried form for up to 6 months. The viability of KID7 cells in SMG combination was high at 4°C followed by −20°C and 25°C (Figure 7B). After 6 months of storage, KID7 cells in SMG matrix retained >90% viability at 4°C and = 89% viability at −20°C, which resulted in 1-log-order decrease in viable cells (Figure 7B) whereas at 25°C the viability was reduced to 68% with 2-log-order decrease in the viable cells (Figure 7B). These results suggest that KID7 combined with SMG matrix could be stored long term at 4°C in freeze-dried form and demonstrate the technological properties of the strain.

## Discussion

*P. pentosaceus* has been approved as an animal feed additive in the United States, European Union, China, Thailand, Australia, and New Zealand (Lim and Tan, 2009). In recent years it has received increased attention and has been characterized for its possible utilization as probiotic in humans (Osmanagaoglu et al., 2012; Zhao et al., 2012; Borges et al., 2013; Vidhyasagar and Jeevaratnam, 2013; Borges and Teixeira, 2014; Garcia-Ruiz et al., 2014; Lee et al., 2014).

Microbes used as probiotics should satisfy certain safety criteria such as being negative for mucin degradation activity (Abe et al., 2010), since microbes degrading mucin can translocate from the intestinal lumen to other body parts and cause bacteremia (Abe et al., 2010). Generally, members of LAB does not possess hemolytic activity. However, *Enterococcus* and *Bacillus* strains possess hemolytic potential and such strains should be adequately tested and confirmed for non-hemolytic activity (FAO-WHO, 2002). Testing for bioamine production is an important safety test, since most of the LAB have the ability to produce bioamines (Bover-Cid and Holzapfel, 1999). These are toxic substances generated from the decarboxylation of amino acids present in foods, the most important being diamines, which are precursors of carcinogenic nitrosamines (Bover-Cid and Holzapfel, 1999). Bover-Cid and Holzapfel (1999) observed that pediococci do not show any ability to produce bioamines, which is in accordance with our observation that KID7 did not produce bioamine on any of the amino acids tested in this study (Figure S2). Antibiotic susceptibility is another key requirement of a probiotic strain used for human or animal consumption (FAO-WHO, 2002; EFSA, 2012). Ingestion of probiotic bacteria with transmissible (plasmid or transposon-borne) antibiotic-resistant genes may lead to generation of new antibiotic-resistant pathogens in the host gut by horizontal gene transfer (EFSA, 2012). However, ingestion of probiotic bacteria containing non-transmissible drug resistance genes pose a low potential for horizontal transfer and generally may be used as feed-additive (EFSA, 2012). The strain KID7 was resistant to kanamycin and streptomycin. *Pediococcus pentosaceus* strains were shown to have MICs of 256 to >256 and up to 192 μg/ml for kanamycin and streptomycin, respectively (Danielsen et al., 2007), which are higher than the EFSA recommended cut-off values. Strains showing higher MIC than the EFSA cut-off values are considered resistant and must be studied for the genetic basis of resistance and must be of non-transmissible nature; otherwise, those strains should not be used as feed additives (EFSA, 2012). The aminoglycoside resistance gene *aac(6*′*)Ie-aph(2*″*)Ia* has been reported in some *Pediococcus* species (Ammor et al., 2007), which codes for the bifunctional aminoglycoside-modifying enzyme 69-N-aminoglycoside acetyltransferase-20-O-aminoglycoside phosphotransferase (Tenorio et al., 2001). The *aac(6*′*)Ie-aph(2*″*)Ia* gene confers high level of resistance to gentamicin in addition to other aminoglycoside antibiotics (Tenorio et al., 2001), however, KID7 was susceptible to gentamicin. The genetic basis of streptomycin and kanamycin resistance in strain KID7 is not known and needs further study to assess the nature of resistance to these aminoglycoside antibiotics.

Several previous reports on *P. pentosaceus* studied the acid and bile tolerance separately (Jonganurakkun et al., 2008; Osmanagaoglu et al., 2010; Vidhyasagar and Jeevaratnam, 2013; Garcia-Ruiz et al., 2014; Lee et al., 2014); however, in real situations, the consumed probiotic should pass through the physiological events of ingestion and digestion of the human gastrointestinal tract (GIT), which include passage through the oral cavity, stomach and the small intestine (Bove et al., 2012). Tolerance to the GIT is one of the selection criterion recommended by FAO-WHO (2002); hence, we studied the tolerance of KID7 to progressive oral, gastric, and intestinal stress (OGT assay) in order to mimic the physiological condition. Tolerance of *L. plantarum* strains to oral and gastric stress up to pH 3 was reported previously (Bove et al., 2012). However, when the pH reduced from 3 to 2 a drastic drop of viability was observed for all *L. plantarum* strains and *L. acidophilus* LA5 (Bove et al., 2012) and at the end of the OGT assay a tendency to recover viability was noticed for bacterial samples (Bove et al., 2012). In our study, we observed KID7 strain was able to recover from cell damage caused by the OGT transit (Figure 1), which is in accordance with Bove et al. (2012). Survival and tolerance of the strains are essential to express the probiotic functions in the intestine (Kumar et al., 2011).

Adherence and colonization of LAB strains in the intestine can be studied using human intestinal epithelial cell models such as Caco-2 and HT-29 cell lines, *in vitro* (Tuo et al., 2013). KID7 showed higher adherence to Caco-2 cells as compared to *L. rhamnosus* GG and *L. acidophilus* ATCC 43121. A good adherence capacity can promote gut residence time of a probiotic bacteria, aids in pathogen exclusion and interaction with host cells for the protection of epithelial cells and immune modulation (Lebeer et al., 2008). Several previous reports demonstrated that *P. pentosaceus* strains adhered to intestinal epithelial cells better than *Lactobacillus* strains (Turpin et al., 2012; Thirabunyanon and Hongwittayakorn, 2013; Garcia-Ruiz et al., 2014).

Antimicrobial activity of LAB against pathogens can be due to the production of organic acids, hydrogen peroxide (H_2_O_2_), and bacteriocins (Vesterlund, 2009). KID7 does not produce H_2_O_2_ (Figure S5). The antimicrobial activity of culture filtrate from KID7 against gram-positive and -negative pathogenic bacteria and not against LAB strains suggest that the antimicrobial activity could be due to the production of organic acids.

BSH enzymes deconjugate the conjugated bile acids into the amino acids (glycine or taurine) and free bile acids (cholic acid or deoxycholic acid) (Joyce et al., 2014). It has been reported that high-level expression of BSH activity by gut microbiota resulted in host weight reduction and cholesterol reduction (Joyce et al., 2014). Hence, BSH activity is an important selection criterion for probiotic microbes to be used for cholesterol reduction. Furthermore, BSH activity is also among the selection criteria recommended by FAO-WHO (2002) for screening potential probiotic strains. Bile acids are synthesized in the liver using cholesterol as precursor. BSH activity of probiotics in the gut could modify the conjugated bile acids and excretion of the free bile acids, which results in *de novo* synthesis of conjugated bile acids from cholesterol to maintain hepatic bile acid pool (Herrema et al., 2010; Degirolamo et al., 2014). Thus, bacterial BSH activity in the gut acts as a sink for cholesterol (Joyce et al., 2014).

Several LAB strains have been reported to possess *in vitro* cholesterol assimilation/uptake activity. LAB exhibit mechanisms, such as cholesterol adsorption to cell wall, and cholesterol assimilation into cell membrane (Liong and Shah, 2005a) and co-precipitation with deconjugated bile acids (Liong and Shah, 2005b) in *in vitro* assays. Cholesterol removal from culture medium by KID7 could involve any of these mechanisms. Previous reports on cholesterol reduction by *Pediococcus* strains showed varying levels (58–73%) of cholesterol reduction in liquid medium supplied with 100–200 μg/ml of cholesterol in the presence of 0.3% bile (Vidhyasagar and Jeevaratnam, 2013). In the present study, we observed a reduction in cholesterol-lowering activity in the presence of 0.3 and 0.5% bile oxgall, dose-dependently (Figure 3B). This reduction in the cholesterol assimilation activity is due to reduction in cell growth of KID7 in 0.3 and 0.5% bile oxgall (Figure 3C). The reduction in cell growth in the presence of oxgall was also reported for a *L. fermentum* strain (Pereira et al., 2003), however, the cholesterol assimilation activity increased despite low growth rate. In our case, lower growth led to lower cholesterol assimilation by KID7 cells. Although, KID7 tolerated bile during brief exposure of 2 h and showed good viability in the OGT assay, growth of the strain was significantly affected during longer exposure to bile as is the case with cholesterol-lowering activity in the presence of bile. The type strain *P. pentosaceus* KACC 12311 also followed the same pattern as KID7; however, it showed relatively lower growth and cholesterol assimilation activity in the presence and absence of bile oxgall. The lower growth of the KID7 and *P. pentosaceus* KACC 12311 in bile-supplied medium could be due to bile salt-deconjugating activity of the strains, which leads to accumulation of free bile acids that are more toxic than conjugated bile (Pereira et al., 2003). However, these *in vitro* assays do not reflect the true nature of probiotic strains in cholesterol-lowering activity, since the conditions *in vivo* are completely different. Hence, the probiotic activity of any strain must be validated through *in vivo* studies.

We tested the strain KID7 *in vivo* to confirm the cholesterol-lowering activity in atherogenic diet-induced hypercholesterolemic mice in comparison with the previously reported cholesterol-lowering probiotic strain *L. acidophilus* ATCC 43121 as positive control. Supplementation of hypercholesterolemic mice with strain KID7 significantly lowered the serum total cholesterol level, LDL level and hepatic total cholesterol level. Reduction in the serum LDL level and cholesterol level correlated with our observation of increased expression of genes related to cholesterol metabolism in liver such as LDLR and ApoE (Figure 6). Increased expression of LDL-receptor will lead to increased absorption of LDL by hepatocytes. The absorbed LDL is degraded and the cholesterol is added to the cholesterol pool (Cohen, 2008). An increase in cholesterol pool will down regulate HMG CoA-reductase (*HMGCR*) and LDL receptor (*LDLR*) expression. In our study, we did not observe any significant down regulation in *HMGCR* expression in the HCD-KID7 group compared to HCD group, whereas *LDLR* expression and *APOE* expression increased in the HCD-KID7 group. This could be due to sinking cholesterol level in cholesterol pool due to expression of *CYP7A1* (Figure 6), which codes for the enzyme cholesterol 7-α-hydroxylase, which catalyzes a rate-limiting step in the *de novo* synthesis of bile acids from cholesterol (Cohen, 2008). Bile salts are secreted into bile duct at a rate of ~24 g/day, but their synthesis is only a fraction of that rate, because most of the bile salts are recycled to the liver from the ileum (Cohen, 2008). Reduction in bile salt reabsorption due to bile salt hydrolase activity in the gut will cause bile synthesis from cholesterol to maintain the bile salts pool size. The increased expression of *CYP7A1* and a decrease in hepatic cholesterol pool also correlated with an increase in total cholic acid excretion in feces of the HCD-KID7 group (Figure 5D). These results suggests that cholesterol-lowering activity of KID7 *in vivo* could be due to its bile salt hydrolase activity. We also observed a significant increase in the level of hepatic LDL mRNA expression in the HCD-*L.ac* group, which is consistent with Park et al. (2007). In addition to the *LDLR* gene, we observed the expression of *SREBF2*, and *APOE* genes in the HCD-*L.ac* group, which was not previously reported in studies using *L. acidophilus* ATCC 43121.

A previous study (Moon et al., 2014) on lipid-lowering effect of *Pediococcus acidilactici* strain M76 and *P. acidilactici* DSM 20284 reported a reduction in serum cholesterol level of 12 and 2.4%, respectively, and a reduction in total hepatic cholesterol level of 30.5 and 14.9%, respectively for the two *P. acidilactici* strains used in their study. Compared to those results, KID7 showed a 19.8% reduction in serum total cholesterol level, a 35.5% reduction in LDL cholesterol level, and a reduction in liver total cholesterol level of 19.7%. Our results indicated that KID7 could be used as a potential cholesterol-lowering probiotic that needs to be confirmed by clinical trials in humans.

Probiotic supplements are usually in the form of dry powders to reduce water activity and protect viability. The preferred drying method is freeze-drying as it preserves viability of the probiotic bacteria. However, not all strains tolerate drying and may show significant loss of viability (Ross et al., 2005). Hence, protective agents and stabilizing agents are added while freeze-drying (Ross et al., 2005). The combination of skim milk and galactomannan increased the viability of KID7 after freeze-drying and subsequent storage conditions. It was reported that skim milk acts as a cryoprotectant and prevents the cells from the damage during freeze-drying (Ross et al., 2005). Additionally, galactomannan from locust bean is a natural polysaccharide used as emulsifying, thickening, and stabilizing agent in the food industry (Cheow et al., 2014). It has been reported elsewhere that spray-drying of probiotic *Lactobacillus paracasei* NFBC in a milk medium supplemented with gum acacia provided better protection to the cells than milk powder alone (Desmond et al., 2002).

In conclusion, the *P. pentosaceus* KID7 reported in this study exhibited cholesterol-lowering activity *in vivo* and possessed the essential characteristics to become a potential probiotic. Furthermore, KID7 maintained good viability during freeze-drying and storage when formulated with a combination of skim milk and galactomannan, which can be utilized in the large-scale production of *P. pentosaceus* KID7-based probiotic products after validation of the probiotic ability in human clinical trials.

### Conflict of interest statement

The authors declare that the research was conducted in the absence of any commercial or financial relationships that could be construed as a potential conflict of interest.

## References

[B1] Abe F. Muto M. Yaeshima T. Iwatsuki K. Aihara H. Ohashi Y.. (2010). Safety evaluation of probiotic bifidobacteria by analysis of mucin degradation activity and translocation ability. Anaerobe 16, 131–136. 10.1016/j.anaerobe.2009.07.006 19638311

[B2] Ahn T. G. Lee J. Y. Cheon S. Y. An H. J. Kook Y. B. (2013). Protective effect of Sam-Hwang-Sa-Sim-Tang against hepatic steatosis in mice fed a high-cholesterol diet. BMC Complement. Altern. Med. 13:366. 10.1186/1472-6882-13-366 24364887 PMC3900264

[B3] Ammor M. S. Flórez A. B. Mayo B. (2007). Antibiotic resistance in non-enterococcal lactic acid bacteria and bifidobacteria. Food Microbiol. 24, 559–570. 10.1016/j.fm.2006.11.001 17418306

[B4] Borges S. Barbosa J. Silva J. Teixeira P. (2013). Evaluation of characteristics of *Pediococcus* spp. to be used as a vaginal probiotic. J. Appl. Microbiol. 115, 527–538. 10.1111/jam.12232 23611355

[B5] Borges S. Teixeira P. (2014). *Pediococcus pentosaceus* SB83 as a potential probiotic incorporated in a liquid system for vaginal delivery. Benef. Microbes 5, 421–426. 10.3920/BM2013.0084 25097107

[B6] Bove P. Gallone A. Russo P. Capozzi V. Albenzio M. Spano G.. (2012). Probiotic features of *Lactobacillus plantarum* mutant strains. Appl. Microbiol. Biotechnol. 96, 431–441. 10.1007/s00253-012-4031-2 22573266

[B7] Bover-Cid S. Holzapfel W. H. (1999). Improved screening procedure for biogenic amine production by lactic acid bacteria. Int. J. Food Microbiol. 53, 33–41. 10.1016/S0168-1605(99)00152-X 10598112

[B8] Chavez M. N. Krone C. L. (1976). Silicic acid thin-layer chromatography of conjugated and free bile acids. J. Lipid Res. 17, 545–547. 965845

[B9] Cheow W. S. Kiew T. Y. Hadinoto K. (2014). Controlled release of *Lactobacillus rhamnosus* biofilm probiotics from alginate-locust bean gum microcapsules. Carbohydr. Polym. 103, 587–595. 10.1016/j.carbpol.2014.01.036 24528770

[B10] Cohen D. E. (2008). Balancing cholesterol synthesis and absorption in the gastrointestinal tract. J. Clin. Lipidol. 2, S1–S3. 10.1016/j.jacl.2008.01.004 19343078 PMC2390860

[B11] Danielsen M. Simpson P. J. O'Connor E. B. Ross R. P. Stanton C. (2007). Susceptibility of *Pediococcus* spp. to antimicrobial agents. J. Appl. Microbiol. 102, 384–389. 10.1111/j.1365-2672.2006.03097.x 17241343

[B12] Degirolamo C. Rainaldi S. Bovenga F. Murzilli S. Moschetta A. (2014). Microbiota modification with probiotics induces hepatic bile acid synthesis via downregulation of the Fxr-Fgf15 axis in mice. Cell Rep. 7, 12–18. 10.1016/j.celrep.2014.02.032 24656817

[B13] Desmond C. Ross R. P. O'callaghan E. Fitzgerald G. Stanton C. (2002). Improved survival of *Lactobacillus paracasei* NFBC 338 in spray-dried powders containing gum acacia. J. Appl. Microbiol. 93, 1003–1011. 10.1046/j.1365-2672.2002.01782.x 12452956

[B14] Ding X. Fan S. Lu Y. Zhang Y. Gu M. Zhang L. (2012). *Citrus ichangensis* peel extract exhibits anti-metabolic disorder effects by the inhibition of PPARγ and LXR signaling in high-fat diet-induced C57BL/6 mouse. Evid. Based Complement. Alternat. Med. 2012:678592. 10.1155/2012/678592 23320036 PMC3536358

[B15] EFSA (2012). Guidance on the assessment of bacterial susceptibility to antimicrobials of human and veterinary importance. EFSA J. 10:2740 10.2903/j.efsa.2012.2740

[B16] FAO-WHO (2002). Joint FAO/WHO Working Group Report on Drafting Guidelines for the Evaluation of Probiotics in Food. Food and Agricultural Organization of the United Nations. Available online at: ftp://ftp.fao.org/es/esn/food/wgreport2.pdf

[B17] Folch J. Lees M. Stanley G. H. S. (1957). A simple method for the isolation and purification of total lipids from animal tissues. J. Biol. Chem. 226, 497–509. 13428781

[B18] Friedewald W. T. Levy R. I. Fredrickson D. S. (1972). Estimation of the concentration of low-density lipoprotein cholesterol in plasma, without use of the preparative ultracentrifuge. Clin. Chem. 18, 499–502. 4337382

[B19] Fuentes M. C. Lajo T. Carrión J. M. Cuñé J. (2013). Cholesterol-lowering efficacy of *Lactobacillus plantarum* CECT 7527, 7528 and 7529 in hypercholesterolaemic adults. Br. J. Nutr. 109, 1866–1872. 10.1017/S000711451200373X 23017585

[B20] García-Ruiz A. González De Llano D. Esteban-Fernández A. Requena T. Bartolomé B. Moreno-Arribas M. V. (2014). Assessment of probiotic properties in lactic acid bacteria isolated from wine. Food Microbiol. 44, 220–225. 10.1016/j.fm.2014.06.015 25084666

[B21] Guardamagna O. Amaretti A. Puddu P. E. Raimondi S. Abello F. Cagliero P.. (2014). Bifidobacteria supplementation: effects on plasma lipid profiles in dyslipidemic children. Nutrition 30, 831–836. 10.1016/j.nut.2014.01.014 24985000

[B22] Guo C. F. Zhang L. W. Han X. Li J. Y. Du M. Yi H. X.. (2011). A sensitive method for qualitative screening of bile salt hydrolase-active lactobacilli based on thin-layer chromatography. J. Dairy Sci. 94, 1732–1737. 10.3168/jds.2010-3801 21426961

[B23] Herrema H. Meissner M. Van Dijk T. H. Brufau G. Boverhof R. Oosterveer M. H.. (2010). Bile salt sequestration induces hepatic *de novo* lipogenesis through farnesoid X receptor and liver X receptor alpha-controlled metabolic pathways in mice. Hepatology 51, 806–816. 10.1002/hep.23408 19998408

[B24] Hill C. Guarner F. Reid G. Gibson G. R. Merenstein D. J. Pot B.. (2014). Expert consensus document: the International Scientific Association for Probiotics and Prebiotics consensus statement on the scope and appropriate use of the term probiotic. Nat. Rev. Gastroenterol. Hepatol. 11, 506–514. 10.1038/nrgastro.2014.66 24912386

[B25] Ishimwe N. Daliri E. B. Lee B. H. Fang F. Du G. (2015). The perspective on cholesterol-lowering mechanisms of probiotics. Mol. Nutr. Food. Res. 59, 94–105. 10.1002/mnfr.201400548 25403164

[B26] Jones M. L. Martoni C. J. Parent M. Prakash S. (2012). Cholesterol-lowering efficacy of a microencapsulated bile salt hydrolase-active *Lactobacillus reuteri* NCIMB 30242 yoghurt formulation in hypercholesterolaemic adults. Br. J. Nutr. 107, 1505–1513. 10.1017/S0007114511004703 22067612

[B27] Jones M. L. Tomaro-Duchesneau C. Prakash S. (2014). The gut microbiome, probiotics, bile acids axis, and human health. Trends Microbiol. 22, 306–308. 10.1016/j.tim.2014.04.010 24836108

[B28] Jonganurakkun B. Wang Q. Xu S. H. Tada Y. Minamida K. Yasokawa D.. (2008). *Pediococcus pentosaceus* NB-17 for probiotic use. J. Biosci. Bioeng. 106, 69–73. 10.1263/jbb.106.69 18691534

[B29] Joyce S. A. Macsharry J. Casey P. G. Kinsella M. Murphy E. F. Shanahan F.. (2014). Regulation of host weight gain and lipid metabolism by bacterial bile acid modification in the gut. Proc. Natl. Acad. Sci. U.S.A. 111, 7421–7426. 10.1073/pnas.1323599111 24799697 PMC4034235

[B30] Kumar R. Grover S. Batish V. K. (2011). Hypocholesterolaemic effect of dietary inclusion of two putative probiotic bile salt hydrolase-producing *Lactobacillus plantarum* strains in Sprague-Dawley rats. Br. J. Nutr. 105, 561–573. 10.1017/S0007114510003740 20923582

[B31] Lebeer S. Vanderleyden J. De Keersmaecker S. C. (2008). Genes and molecules of lactobacilli supporting probiotic action. Microbiol. Mol. Biol. Rev. 72, 728–764. 10.1128/MMBR.00017-08 19052326 PMC2593565

[B32] Lee K. W. Park J. Y. Sa H. D. Jeong J. H. Jin D. E. Heo H. J.. (2014). Probiotic properties of *Pediococcus* strains isolated from jeotgals, salted and fermented Korean sea-food. Anaerobe 28, 199–206. 10.1016/j.anaerobe.2014.06.013 24979684

[B33] Lim A. Tan H.-M. (2009). Probiotics: legal status and regulatory issues section 1.8.2. animal probiotics in Handbook of Probiotics and Prebiotics, eds Lee Y. K. Salminen S. (Hoboken, NJ: John Wiley & Sons, Inc.). 123–139.

[B34] Liong M. T. Shah N. P. (2005a). Acid and bile tolerance and cholesterol removal ability of *Lactobacilli* strains. J. Dairy Sci. 88, 55–66. 10.3168/jds.S0022-0302(05)72662-X 15591367

[B35] Liong M. T. Shah N. P. (2005b). Bile salt deconjugation ability, bile salt hydrolase activity and cholesterol co-precipitation ability of lactobacilli strains. Int. Dairy J. 15, 391–398. 10.1016/j.idairyj.2004.08.007

[B36] Marteau P. Minekus M. Havenaar R. Huis in't Veild J. H. (1997). Survival of lactic acid bacteria in a dynamic model of the stomach and small intestine: validation and the effects of bile. J. Diary Sci. 80, 1031–1037. 10.3168/jds.S0022-0302(97)76027-2 9201571

[B37] Mcdonald L. C. Mcfeeters R. F. Daeschel M. A. Fleming H. P. (1987). A differential medium for the enumeration of homofermentative and heterofermentative lactic acid bacteria. Appl. Environ. Microbiol. 53, 1382–1384. 16347367 10.1128/aem.53.6.1382-1384.1987 PMC203874

[B38] Moon Y. J. Baik S. H. Cha Y. S. (2014). Lipid-lowering effects of *Pediococcus acidilactici* M76 isolated from Korean traditional makgeolli in high fat diet-induced obese mice. Nutrients 6, 1016–1028. 10.3390/nu6031016 24609135 PMC3967175

[B39] Nagpal R. Kumar A. Kumar M. Behare P. V. Jain S. Yadav H. (2012). Probiotics, their health benefits and applications for developing healthier foods: a review. FEMS Microbiol. Lett. 334, 1–15. 10.1111/j.1574-6968.2012.02593.x 22568660

[B40] Osmanagaoglu O. Kiran F. Ataoglu H. (2010). Evaluation of *in vitro* probiotic potential of *Pediococcus pentosaceus* OZF isolated from human breast milk. Probiotics Antimicrob. Proteins 2, 162–174. 10.1007/s12602-010-9050-7 26781239

[B41] Osmanagaoglu O. Kiran F. Yagci F. C. Gursel I. (2012). Immunomodulatory function and *in vivo* properties of *Pediococcus pentosaceus* OZF, a promising probiotic strain. Ann. Microbiol. 63, 1311–1318. 10.1007/s13213-012-0590-9

[B42] Park Y. H. Kim J. G. Shin Y. W. Kim S. H. Whang K. Y. (2007). Effect of dietary inclusion of *Lactobacillus acidophilus* ATCC 43121 on cholesterol metabolism in rats. J. Microbiol. Biotechnol. 17, 655–662. 18051279

[B43] Pavlovic N. Stankov K. Mikov M. (2012). Probiotics interactions with bile acids and impact on cholesterol metabolism. Appl. Biochem. Biotechnol. 168, 1880–1895. 10.1007/s12010-012-9904-4 23054820

[B44] Pereira D. I. Mccartney A. L. Gibson G. R. (2003). An *in vitro* study of the probiotic potential of a bile-salt-hydrolyzing *Lactobacillus fermentum* strain, and determination of its cholesterol-lowering properties. Appl. Environ. Microbiol. 69, 4743–4752. 10.1128/AEM.69.8.4743-4752.2003 12902267 PMC169108

[B45] Ross R. P. Desmond C. Fitzgerald G. F. Stanton C. (2005). Overcoming the technological hurdles in the development of probiotic foods. J. Appl. Microbiol. 98, 1410–1417. 10.1111/j.1365-2672.2005.02654.x 15916653

[B46] Saito M. Seki M. Iida K.-I. Nakayama H. Yoshida S.-I. (2007). A novel agar medium to detect hydrogen peroxide-producing bacteria based on the prussian blue-forming reaction. Microbiol. Immunol. 51, 889–892. 10.1111/j.1348-0421.2007.tb03971.x 17895606

[B47] Stackebrandt E. (2011). Molecular taxonomic parameters. Microbiol. Aust. 2, 59–61.

[B48] Sultan S. Hynes N. (2013). The ugly side of statins. Systemic appraisal of the contemporary un-known unknowns. Open J. Endocr. Metab. Dis. 03, 179–185. 10.4236/ojemd.2013.33025

[B49] Tamura K. Stecher G. Peterson D. Filipski A. Kumar S. (2013). MEGA6: molecular evolutionary genetics analysis version 6.0. Mol. Biol. Evol. 30, 2725–2729. 10.1093/molbev/mst197 24132122 PMC3840312

[B50] Tenorio C. Zarazaga M. Martinez C. Torres C. (2001). Bifunctional enzyme 6′-*N*-aminoglycoside acetyltransferase-2”-O- aminoglycoside phosphotransferase in *Lactobacillus* and *Pediococcus* isolates of animal origin. J. Clin. Microbiol. 39, 824–825. 10.1128/JCM.39.2.824-825.2001 11281121 PMC87833

[B51] Thirabunyanon M. Hongwittayakorn P. (2013). Potential probiotic lactic acid bacteria of human origin induce antiproliferation of colon cancer cells via synergic actions in adhesion to cancer cells and short-chain fatty acid bioproduction. Appl. Biochem. Biotechnol. 169, 511–525. 10.1007/s12010-012-9995-y 23239414

[B52] Trautvetter U. Ditscheid B. Kiehntopf M. Jahreis G. (2012). A combination of calcium phosphate and probiotics beneficially influences intestinal lactobacilli and cholesterol metabolism in humans. Clin. Nutr. 31, 230–237. 10.1016/j.clnu.2011.09.013 22019281

[B53] Tuo Y. Yu H. Ai L. Wu Z. Guo B. Chen W. (2013). Aggregation and adhesion properties of 22 *Lactobacillus* strains. J. Dairy Sci. 96, 4252–4257. 10.3168/jds.2013-6547 23664349

[B54] Turpin W. Humblot C. Noordine M. L. Thomas M. Guyot J. P. (2012). *Lactobacillaceae* and cell adhesion: genomic and functional screening. PLoS ONE 7:e38034. 10.1371/journal.pone.0038034 22675431 PMC3364998

[B55] Vesterlund S. (2009). Mechanisms of probiotics: production of antimicrobial substances, in Handbook of Probiotics and Prebiotics, eds Lee Y. K. Salminen S. (Hoboken, NJ: JohnWiley & Sons, Inc.).

[B56] Vidhyasagar V. Jeevaratnam K. (2013). Evaluation of *Pediococcus pentosaceus* strains isolated from Idly batter for probiotic properties *in vitro*. J. Func. Foods 5, 235–243. 10.1016/j.jff.2012.10.012

[B57] Wang H. Chen G. Ren D. Yang S. T. (2014). Hypolipidemic activity of Okra is mediated through inhibition of lipogenesis and upregulation of cholesterol degradation. Phytother. Res. 28, 268–273. 10.1002/ptr.4998 23606408

[B58] Wang J. Yuan X. Sun B. Tian Y. Cao Y. (2009). Scavenging activity of enzymatic hydrolysates from wheat bran. Food Technol. Biotechnol. 47, 39–46.

[B59] Wiegand I. Hilpert K. Hancock R. E. (2008). Agar and broth dilution methods to determine the minimal inhibitory concentration (MIC) of antimicrobial substances. Nat. Protoc. 3, 163–175. 10.1038/nprot.2007.521 18274517

[B60] Zhao X. Higashikawa F. Noda M. Kawamura Y. Matoba Y. Kumagai T.. (2012). The obesity and fatty liver are reduced by plant-derived *Pediococcus pentosaceus* LP28 in high fat diet-induced obese mice. PLoS ONE 7:e30696. 10.1371/journal.pone.0030696 22363472 PMC3281851

[B61] Zhou J. S. Gopal P. K. Gill H. S. (2001). Potential probiotic lactic acid bacteria *Lactobacillus rhamnosus* (HN001), *Lactobacillus acidophilus* (HN017) and *Bifidobacterium lactis* (HN019) do not degrade gastric mucin *in vitro*. Int. J. Food Microbiol. 63, 81–90. 10.1016/S0168-1605(00)00398-6 11205957

