# Supplementary material for: Preliminary probiotic and technological characterization of Pediococcus pentosaceus strain KID7 and in vivo assessment of its cholesterol-lowering activity
Source: Front Microbiol. 2015 Aug 4;6:768. doi: 10.3389/fmicb.2015.00768 (PMC4523826; doi:10.3389/fmicb.2015.00768)
Supplement: Supplementary file 1 [file Presentation1.PDF]

Supplementary Material

Manuscript Submitted to Frontiers in Microbiology

Preliminary probiotic and technological characterization of *Pediococcus pentosaceus* strain KID7 and *in vivo* assessment of its cholesterol-lowering activity

Karthiyaini Damodharan,[1,2,†] Young Sil Lee,[2,†] Sasikumar Arunachalam Palaniyandi,[2,3,†] Seung Hwan Yang,[2,3,*] and Joo-Won Suh[1,2,*]

[1]Division of Bioscience and Bioinformatics, [2]Center for Nutraceutical and Pharmaceutical Materials, [3]Graduate School of Interdisciplinary program of Biomodulation, College of Natural Science, Myongji University, Cheoin-gu, Yongin, Gyeonggi-Do 449-728, Korea.

[*] Correspondence

SH Yang: Phone: 82-31-330-6880; Fax: 82-31-336-0870

E-mail: ymichigan@mju.ac.kr

JW Suh: Phone: 82-31-330-6190; Fax: 82-31-336-0870

E-mail: jwsuh@mju.ac.kr

[†]These authors contributed equally to this work

Short Title: Probiotic characterization of *Pediococcus pentosaceus* KID7

Table S1 Phenotypic characteristics of *Pediococcus pentosaceus* strain KID7

Phenotypic characteristics	KID7	*P. pentosaceus* KACC 12311
Gram's stain	+	+
Catalase test	-	-
**Carbohydrate fermentation**¶		
L-arabinose	-	+
Ribose	+	+
D-xylose	-	+
Galactose	+	+
Glucose	+	+
Fructose	+	+
Mannose	+	+
Dulcitol	-	+
Sorbitol	-	+
N-acetyl-Glucosamine	±	+
Amygdalin	-	+
Arbutin	±	+
Esculin	+	+
Salicin	-	+
Cellobiose	+	+
Maltose	+	+
Lactose	-	+
Mellibiose	-	+
Sucrose	+	+
Trehalose	+	+
Raffinose	+	±
Gentobiose	+	±
D-tagatose	+	+
Gluconate	+	±
**Enzyme activity**#		
β-galactosidase activity	+	+
β-glucosidase activity	+	+
Protease activity	+	+
**Fermentation pattern**$	Homo-fermentation	Homo-fermentation

¶Carbohydrate fermentation pattern of KID7 in API50 CHL kit; +, positive carbohydrate utilization; ±, moderate utilization; -, no utilization

#+, positive enzyme activity; -, negative enzyme activity

$Green colour colonies on HHD medium indicates homo fermentative pattern

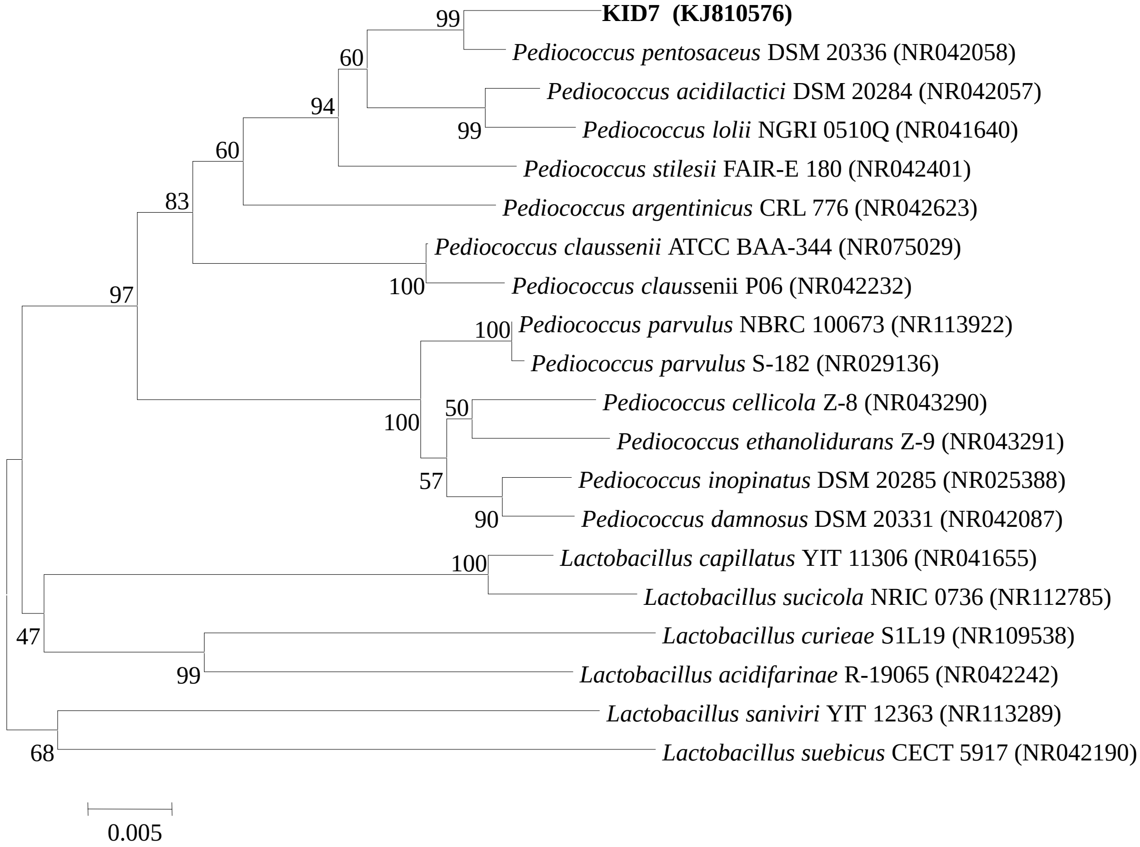

Fig. S1 Phylogenetic tree constructed based on 16S rRNA gene sequence of strain KID7. The strain possess 99% similarity with *P. pentosaceus* DSM 20336.

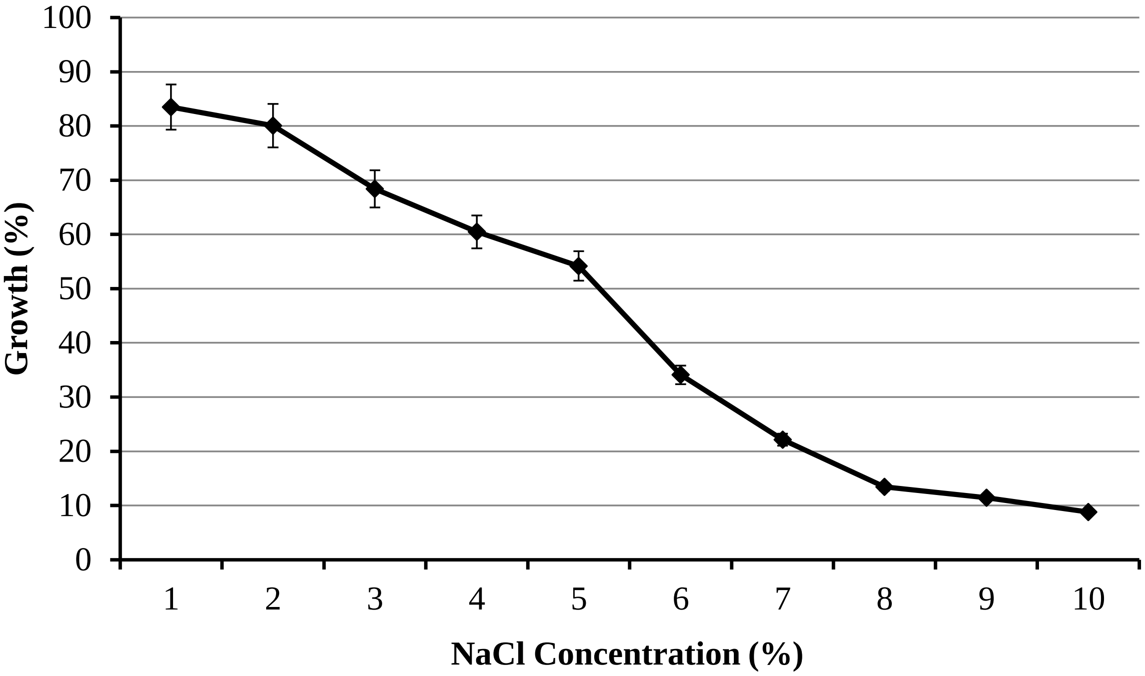

Fig. S2 Growth of strain KID7 in the presence of increasing concentrations of NaCl. Growth of the strain KID7 is presented as percentage of growth relative to growth of the strain in the absence of NaCl. The growth experiment was performed using MRS broth. The error bars presented as ±standard deviation of mean values of three independent experiments, n = 3.

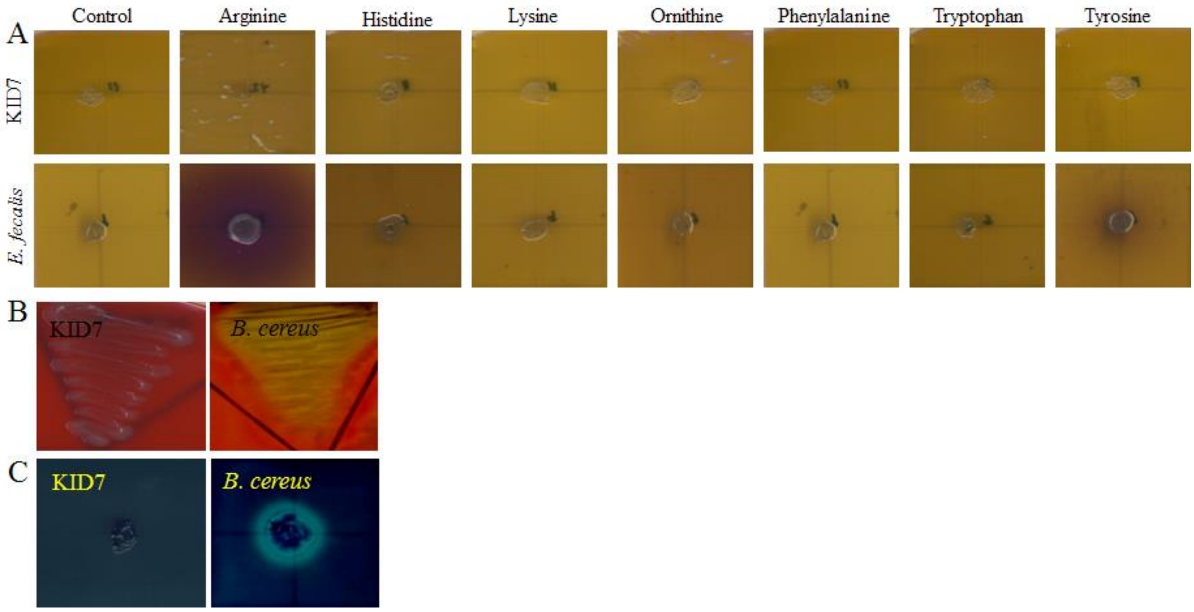

Fig. S3 *In vitro* safety assessment of strain KID7. (**A)** Pictures showing no bioamine production by strain KID7 on various amino acid supplied decarboxylase agar medium; Positive control *Enterococcus faecalis* shows bioamine production in ornithine and arginine supplied medium. (**B)** Picture showing no hemolytic activity of strain KID7; Positive control: *Bacillus cereus* KACC 11240. (**C)** Pictures showing no mucin degradation by KID7, whereas mucin degradation by positive control strain *Bacillus cereus* KACC 11240.

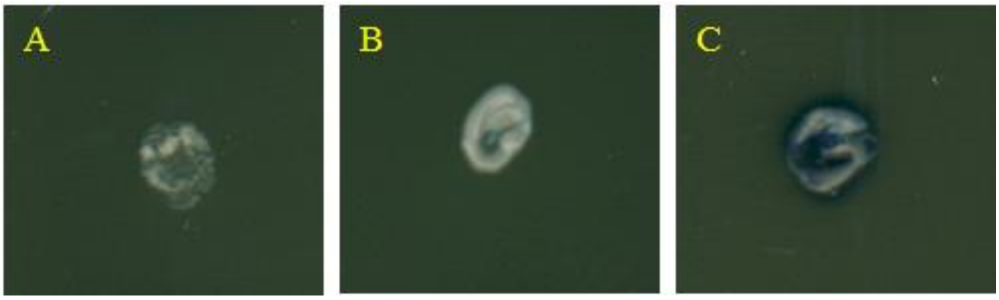

Fig. S4 Hydrogen peroxide production test using Prussian blue (PB) agar. (**A)** Picture showing no colour change in PB agar medium, indicates no hydrogen peroxide production by KID7; (**B)** Picture showing no colour change in PB agar medium, indicates no hydrogen peroxide production by *P. pentosaceus* KACC 12311; (**C)** Picture showing blue colour around the colony in PB agar medium, indicates hydrogen peroxide production by *L. rhamnosus* GG (positive control).
